# Rheumatic heart disease in The Gambia: clinical and valvular aspects at presentation and evolution under penicillin prophylaxis

**DOI:** 10.1186/s12872-021-02308-8

**Published:** 2021-10-18

**Authors:** Lamin E. S. Jaiteh, Lamin Drammeh, Suzanne T. Anderson, John Mendy, Samba Ceesay, Umberto D’Alessandro, Jonathan Carapetis, Mariana Mirabel, Annette Erhart

**Affiliations:** 1 Medical Research Council Unit The Gambia at the London School of Hygiene and Tropical Medicine, Banjul, The Gambia; 2 Edwards Francis Small Teaching Hospital, Ministry of Health, Banjul, The Gambia; 3 Department of Community Child Health, Evelina London, Guys and St Thomas’ NHS Trust, London, England, UK; 4 Acting Director of Health Services, Ministry of Health, Banjul, The Gambia; 5 Telethon Kids Institute, University of Western Australia and Perth Children’s Hospital, Nedlands, Australia; 6 Institut National de la Santé et ed la Recherche Médical (INSERM), U970, 56 rue Leblanc, 75015 Paris, France; 7 Cardio-Oncology, Assistance Publique- Hopitaux de Paris (AP-HP) Centre, University of Paris, 20 rue Leblanc, 75737 Paris, France

**Keywords:** Rheumatic heart disease, The Gambia, Case review, Penicillin prophylaxis, Clinical presentation, Echocardiography, Evolution

## Abstract

**Background:**

Rheumatic heart disease (RHD) remains the leading cause of cardiac-related deaths and disability in children and young adults worldwide. In The Gambia, the RHD burden is thought to be high although no data are available and no control programme is yet implemented. We conducted a pilot study to generate baseline data on the clinical and valvular characteristics of RHD patients at first presentation, adherence to penicillin prophylaxis and the evolution of lesions over time.

**Methods:**

All patients registered with acute rheumatic fever (ARF) or RHD at two Gambian referral hospitals were invited for a clinical review that included echocardiography. In addition, patients were interviewed about potential risk factors, disease history, and treatment adherence. All clinical and echocardiography information at first presentation and during follow-up was retrieved from medical records.

**Results:**

Among 255 registered RHD patients, 35 had died, 127 were examined, and 111 confirmed RHD patients were enrolled, 64% of them females. The case fatality rate in 2017 was estimated at 19.6%. At first presentation, median age was 13 years (IQR [9; 18]), 57% patients had late stage heart failure, and 84.1% a pathological heart murmur. Although 53.2% of them reported history of recurrent sore throat, only 32.2% of them had sought medical treatment. A history suggestive of ARF was reported by 48.7% patients out of whom only 15.8% were adequately treated. Two third of the patients (65.5%) to whom it was prescribed were fully adherent to penicillin prophylaxis. Progressive worsening and repeated hospitalisation was experienced by 46.8% of the patients. 17 patients had cardiac surgery, but they represented only 18.1% of the 94 patients estimated eligible for cardiac surgery.

**Conclusion:**

This study highlights for the first time in The Gambia the devastating consequences of RHD on the health of adolescents and young adults. Our findings suggest a high burden of disease that remains largely undetected and without appropriate secondary prophylaxis. There is a need for the urgent implementation of an effective national RHD control programto decrease the unacceptably high mortality rate, improve case detection and management, and increase community awareness of this disease.

## Introduction

Rheumatic heart disease (RHD) remains the leading cause of cardiac-related deaths and disability in children and young adults worldwide, with global estimates of more than 34 million people affected and > 345,000 annual deaths, mainly in Sub-Saharan Africa [1–3]. RHD is a chronic complication of acute rheumatic fever (ARF), an exaggerated immune response to Group A *Streptococcus* infections, mostly of the throat, occuring usually during childhood and adolescence [4, 5]. RHD complications include heart failure, atrial fibrillation, infective endocarditis, cardio-embolic stroke and adverse pregnancy outcomes [3, 6]. Secondary prophylaxis with 4-weekly injection of benzathine penicillin G (BPG) remains the most effective intervention for preventing RHD progression [7]. However, there are numerous and poorly understood barriers to effective control programmes [8].

RHD thrives in poor, over-crowded communities in low- and middle-income countries (LMICs) with limited access to basic social amenities, including housing and health care [1–3]. Despite its high morbidity and mortality, RHD became largely neglected after it was virtually eliminated in high-income countries (HICs) by the end of the twentieth century [9]. In May 2018, following years of campaigning and advocacy from the RHD community [10] the World Health Organisation (WHO) launched the “Global Resolution on Rheumatic Fever and Rheumatic Heart Disease” which was unanimously adopted by member states [11]. This resolution calls for action to strengthen primary and secondary prevention of ARF and RHD, integrate RHD services into primary health care, secure a reliable supply of BPG, and ensure a well-resourced and trained health workforce to provide RHD services. Indeed, the main barriers to RHD prevention, control and elimination in endemic countries are: the neglect of ARF and RHD in national health policies and budgets; the paucity of data to enable targeting of prevention efforts; poor primary and secondary prevention and access to primary health care; inadequate numbers and training of health workers at all levels; limited understanding of ARF/RHD in affected communities, and inaction on the social determinants of the disease and inequities in health [11]. Although further research in some areas is needed, the biggest gap in RHD control is in the implementation of effective primary and secondary preventive measures. On 6th January 2020, the World Heart Federation (WHF) President met with the WHO Director General to share ambitions and actions which would lead to the elimination of RHD in a generation [11, 12].

In The Gambia, the burden of RHD is thought to be high given the substantial number of children presenting at referral hospitals at an advanced stage of the disease. Indeed, there are more than 250 children with RHD currently being managed as outpatients at the paediatrics department of the Edward Francis Small Teaching Hospital (EFSTH), the main referral and only teaching hospital in The Gambia, and at the Clinical Services Department (CSD) of the Medical Research Council Unit The Gambia at the London School of Hygiene and Tropical Medicine (MRCG). Despite this, no high-quality data are available and no control programme is currently implemented.

In both institutions, care for children with RHD (including consultation, laboratory, imaging and medications) is provided free of charge. Echocardiography is available at the MRCG and is provided free of charge for children while adults are charged ~ US$4. There are three private echocardiography service centres, all of them based at the coast near the capital city, whose services are charged $30 to $40 per patient.

As a first step towards larger, collaborative, multi-disciplinary research projects between the Gambian Health Services and the MRCG to inform the development of an effective national RHD control programme, we conducted a baseline study to review all patients registered as having ARF or RHD at the MRCG and/or EFSTH. The objective was to describe the clinical and valvular characteristics of known RHD patients at first presentation, at last evalution during the present study, adherence to penicillin prophylaxis and the evolution of clinical and valvular lesions over time.

## Methods

### Study design and patients recruitment

This case series analysis was carried out between November 2017 and January 2018 and included children and adult patients (all age) with ARF or RHD registered at two referal hospitals in the Gambia, *i.e.* the MRCG CSD in Fajara or the paediactrics department of the EFSTH in Banjul. A list of patients currently or previously (within the last 3 years) treated for ARF or RHD was generated and individual patients contacted (or their parents/guardians for children < 18) by telephone. The MRCG has an electronic medical records system (EMRS) which enabled a quick search using ARF or RHD as keyword for provisional/definitive diagnosis. At the EFSTH, the clinical records were searched manually. Patients were called individually by the study nurse who explained the project’s objectives and invited them to participate in the study. Several call attempts were made before declaring a patient unreachable. Patients were invited based on their availability, to attend the MRCG CSD for clinical evaluation.

### Data collection

Written informed consent was obtained from all participants, or their parents/guardians for children < 18 years, after detailed information on the study objectives and procedures was provided by the study team in English or one of the local languages (Mandinka, Wolof, Fula). Children aged 12–17 years were invited to provide their written assent. A questionnaire was administered on socio-demographic characteristics, past medical history, including signs and symptoms at first presentation, treatments received and current symptoms, *e.g.* shortness of breath at rest or on exertion. Patients’ medical records (when available) were also searched to extract past medical history.

Vital signs at rest, *i.e.* heart- and respiratory rates, blood pressure (Omron sphygmomanometer), axillary temperature (digital thermometer), oxygen saturation (pulse oximeter), were measured by the study nurse; and a detailed physical examination was perfomed by a medical doctor, including cardio-thoracic auscultation and assessment for signs of right heart failure (hepatomegaly, lower limb oedema). Heart failure was classified into four categories according to the Modified Ross Score for children or the New York Heart Association in adults [13].

Until April 2013, secondary prophylaxis for RHD in The Gambia consisted of monthly injections of Benzathine Penicillin G (BPG) until the age of 20 years for patients with valvular lesions. This was changed to daily oral penicillin V (250 mg BID for children < 30 kg or 500 mg if > 30 kg) following a few sudden deaths shortly after BPG injection that suggested an anaphylactic reaction and/or sub-standard quality drugs [14, 15]. The duration of secondary prophylaxis with penicillin V was unchanged until the time of our study.

### Echocardiography protocols and image interpretation

Each patient was subjected to a 2D, M-mode, colour and spectral Doppler transthoracic echocardiography by the study cardiologist using a Vivid IQ (General Electrics) portable echo machine. Multifrequency phase array transducers (paediactric and adult) with a frequency range of 3—6 MHz were used. A 6-image acquisition protocol was used with focus on valve pathology & function as well as cardiac chamber size and function (atria and ventricles). This protocol included the parasternal long- and short axes views, apical four-chamber, two-chamber & three-chamber views as well as subcostal long axis view in 2D and colour Doppler. In addition, continuous wave Doppler across the mitral and tricuspid inflow, and aortic and pulmonary outflow were measured if any of these valves showed evidence of regurgitation or stenosis. RHD was classified as bordeline or definite (mild, moderate or severe) according to the severity of the most affected valve following the WHF consensus criteria [16]. External quality control of the echocardiography readings was done by an independent cardiologist at INSERM, Paris, France. All anonymised images were shared via the cloud using the DICOM platform (https://www.postdicom.com/). Discrepant results (difference in either diagnosis and/or in severity of the lesion at any of the four valves) were reviewed and discussed by the two readers (via email) until agreement was found. The final data analysis included only patients with confirmed RHD. Cases with normal echocardiography, non-RHD cardiac disease, or other alternative diagnoses were excluded.

### Data analysis

Socio-demographic, clinical and echocardiography data were entered and cleaned in a REDCap online database, and analysed in STATA 12. Summary statistics were computed and differences were tested for statistical significance (p < 0.05) using Student t-test for continuous variables and Chi-square or Fisher Exact accordingly for categorical variables.

Socio-economic status (SES) was categorized as high, medium or low according to the following facilities/assets the patient’s household had or owned: pipewater, gas, electricity, fridge, TV, motorbike, vehicle (tractor, truck, car). The lowest SES category included households that had none of the above mentioned items while the highest category included households with at least a vehicle regardless of the other items; the medium SES category included all the remaining households. We included all forms of Western education (French/English/German, etc.) recognized as formal education in The Gambia but excluded Arabic/Quranic schooling.

Treatment adherence since first presentation was categorized according to the patient’s (and/or parent/guardian’s) own report and his/her medical records as: (i) full (= regular intake with rare interruption); (ii) partial (= regular intake with occasional interruptions), (iii) poor (irregular intake), (iv) and very poor (very irregular intake with prolonged (> 1 week) voluntary interruption). Patients who had deliberately stopped penicillin prophylaxis for more than one month at the time of the study visit were categorized as defaulters. No attempt was made to verify treatment adherence using pill counts or other measures. We tested if adherence was significantly associated with any potential risk factor (i.e., age at presentation, sex, SES, mother’s education, follow-up time, household crowdedness, or the Ross score at presentation) using Fisher Exact- or Chi-square test accordingly (signficant p-value < 0.05). For this purpose, continous variables were categorized (i.e., age categories: 0–10; 11–19; 20+ years; and length of follow-up categories: < 5-, 5–9; 10+ years).

The evolution of valvular lesions (for patients who had a report on their first echocardiography and not undergone cardiac surgery) were assessed and categorized as follows: (i) no change (same lesions and severity as in 1st echo-); (ii) initial lesions decreased (current lesions were less severe or disappeared compared to 1st echo-); (iii) initial lesions worsened (new lesions appeared and/or initial lesions increased in severity).

The association between valvular or clinical evolution and potential risk factors was explored using Fisher Exact- or Chi-square test accordingly (patients with surgery were excluded from this analysis). Significant trends were tested using the z-test for trend.

We estimated the number of reviewed cases in need of cardiac surgery based on the severity of the valvular lesions (severe mitral stenosis and/or regurgitation, severe aortic stenosis and/or regurgitation, severe tricuspid regurgitation—mostly functional) and other echocardiographic findings such as reduced left ventricular systolic function (LVEF < 40%) and severe secondary pulmonary hypertension (Estimated Pulmonary Arterial Systolic Pressure > 60 mmHg) [17–20].

### Ethical considerations

Ethical approval was obtained from the Gambia Government/MRC Joint Ethics Committee (L2017.56). Permissions were given by the Gambian Health Services, the EFSTH in Banjul and the CSD of the MRCG in Fajara.

## Results

### Patients’ recruitment and RHD mortality

**Fig. 1:**
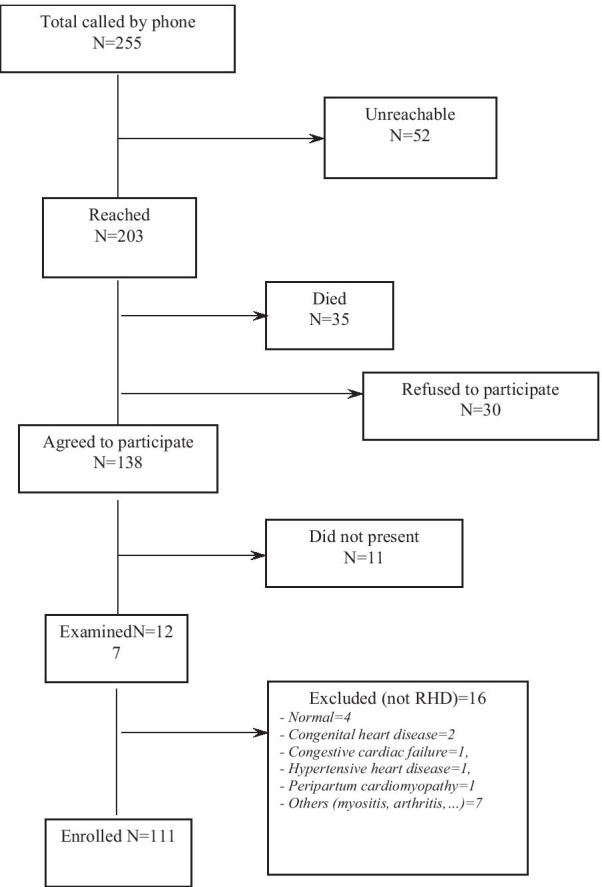
Flowchart showing the recruitment of participants

Among the 255 eligible patients with provisional diagnosis of RHD, 52 (20%) could not be traced, 35 (13.7%) had died, 30 (11.8%) refused to participate, and 11 (4.3%) did not attend the scheduled visit (Fig. 1). Among the 127 patients examined, 16 were excluded from the analysis as they did not meet the RHD case definition, and 111 patients with confirmed RHD by standard echocardiography were enrolled (Fig. 1). The medical records of the deceased patients were reviewed and most of them (30/35 = 85.7%) were ≤ 25 years old with similar numbers of males and females (sex ratio M/F 18/17 = 1.1). The majority died in 2017 (27/35 = 77%) while admitted for heart failure. The case fatality rate in 2017 was estimated at 19.6% (27/138) when considering the 111 RHD patients plus the deceased (N = 111 + 27 = 138).

### Baseline socio-demographic data

Between November 6th, 2017 and January 3rd, 2018, a total of 111 patients (100 at MRCG and 11 at the EFSTH) were reviewed clinically and echographically and confirmed RHD. The majority were females (64%) and the median age was 16 years (IQR [13; 24]). Most patients (77.5%) were either in- or had some level of formal schooling but the majority (65.8%) had a mother without formal education (Table 1). About a fifth of the patients (21.6%) belonged to the poorest socio-economic category, and households were generally crowded with a median of 9 dwellers (IQR [6; 14]). Very few patients slept alone in a room (median of 2 additional persons) or in a bed (median of 1 additional person) (Table 1).

**Table 1:** Socio-demographic characteristics of study patients

Characteristics (N = 111)	N	%
Gender		
Males	40	36
Females	71	64
Age, median [IQR]	16 [13;24]	
Residence		
Costal districts (Kombos, Banjul, Kanifing, Barra town)	97	87.4
Others (included 1 Casamance, 2Fara, 1 Basse, 1Bansang	14	12.6
Moved to Kombos since diagnosis	10	9
Ethnic groups		
Mandinka	49	44.1
Wolof	12	10.8
Fula	14	12.6
Jola	17	15.3
Others (Sarahule, Manjago, Serere)	19	17.1
Education level		
Currently following formal schooling	50	45.1
Previous primary schooling	20	18
Previous secondary schooling	16	14.4
Exclusive Arabic/Quranic schooling	9	8.1
Never had any schooling	16	14.4
Education level of the mother		
None	15	13.5
Primary school	20	18
Secondary school	18	16.2
Exclusive Arabic/Quranic schooling	58	52.3
Socio economic status		
Poorest	24	21.6
Medium	48	43.2
Higher	39	35.1
*Patients’ households crowdeness*		
Persons per households, median [IQR]	9 [6;14]	
Patients’ sleeping room arrangements		
Patient sleeps alone in the room	6	5.4
Median number of other individuals sleeping in the room, [IQR]	2 [1;4]	
Patients’ bed sleeping arrangements		
Patient sleeps alone in his bed	9	8.1
Median number of other individuals sleeping in the bed, [IQR]	1 [1;2]	

### Disease history (Table 2)

**Table 2:** Clinical characteristics at first presentation (Day 0)

Characteristics	N	%
Median follow-up time (months since 1st presentation, N = 87) [IQR]	33 [3;121]	
Median age, [IQR]	13 [9;18]	
History of recurrent sore throat		
Yes	59	53.2
No	52	46.8
Usual treatment for sore throats (N = 59)		
No treatment at all	11	18.6
Traditional medicine (hot water w. salt/lime/pepper,…)	27	45.8
Self-treatment (buy medicines, don’t know what)	2	3.4
Consulted health facilities	19	32.2
Reported history of acute rheumatic fever (ARF)	54	48.7
Treatment for ARF (N = 54)	38	70.4
Consulted health facilities	15	27.8
Traditional treatment	1	1.8
No treatment		
History of skin sores		
Yes	20	18
No	91	82
Modified Ross or NYHA Score (N = 107)		
Class 1 (no dyspnea)	10	9.3
Class 2 (dyspnea, fatigue on moderate exertion)	36	33.6
Class 3 (dyspnea, fatigue on minimal exertion)	22	20.6
Class 4 (dyspnea at rest)	39	36.4
Oedema lower limbs (N = 107)		
No	61	57
Yes (30% Grade 4)	46	43
Heart murmur at auscultation (N = 107)		
No	14	13.1
Yes	90	84.1
Missing	3	2.8
Enlarged liver (N = 107)		
No	63	58.9
Yes	19	17.8
Missing	25	23.4
Penicillin prophylaxis started upon RHD diagnosis		
No	17	15.3
Yes	93	83.8
Missing	1	0.9
Anti-heart failure treatment needed at first presentation		
No	23	20.7
Yes	86	77.5
Missing	2	1.8

The median follow-up time since first presentation was 33 months (IQR[3;121]) ranging from 1 month to 12 years. The median age at first presentation was 13 years (IQR [9; 18]) though 30 patients (27.0%) were first diagnosed with RHD at adult age, among whom 21 were women diagnosed only after up to 9 vaginal deliveries as they went into heart failure. Their first echocardiogram showed severe valvular lesions often associated with pulmonary hypertension.

About half of the 111 patients (53.2%) reported a history of recurrent sore throat for which only 19 (32.2%) had attended a health facility. Most patients did either nothing or used traditional medicine, usually drinking hot water with lime and pepper or with salt. Moreover, about half of the patients (48.7%) reported one or more episodes of fever associated with joint pain and swelling compatible with ARF. Although the majority (38/54, 70.4%) attended a primary or secondary health care facility, only 6 (15.8%) were actually diagnosed and treated for ARF while the others received painkillers and antibiotics. Recurrent skin sores (*e.g.* impetigo, folliculitis) were less commonly reported (< 20% of the patients) but generally medically attended.

### Clinical and valvular characteristics at first presentation

Among the 107 patients with available data from first presentation, 61 (57%) had symptoms of late stage heart failure (Modified Ross/NYHA score 3–4) and a third (33.6%) had a score of 2 (Table 2). Most patients (84.1%) had a pathological heart murmur at auscultation, mainly a grade III–IV pan-systolic murmur at mitral area, and 43% presented with lower limb oedema of whom 30% with anasarca (Table 2). A written report on baseline echocardiography was available for 93 patients among whom 86 had some measured parameters to be compared with those measured during our review (Table 4). On their first echocardiogram, all but five patients had thickened valves (MV) with restricted mobility and/or a prolapse of the anterior mitral valve leaflet (Table 4). Most patients (80/86 = 93.0%) had MV regurgitation, mainly severe (58.8%), while 22 (22/86 = 25.6%) had mitral stenosis, mostly severe (68.2%), and generally associated with mitral valve regurgitation (MVR; 17/22 = 77.3%). More than half of the patients (54.7%) had aortic regurgitation including three with both aortic regurgitation and stenosis. TV regurgitation was common (63/86 = 73.3%) though mostly functional (due to annular dilatation) while pulmonary valve (PV) regurgitation was less commonly found (17/86 = 19.8%). More than a third (33/86 = 38.4%) of the patients had pulmonary hypertension at first presentation and 8% had impaired left ventricular systolic function.

### Penicillin prophylaxis and adherence

The majority of patients (n = 93) were started on penicillin prophylaxis (PP) upon RHD diagnosis (i.e., monthly BPG injections before April 2013 (n = 25) and later, twice daily oral penicillin V (n = 68); Table 2). At the time of our review, 22 patients (23.7%) were no longer under PP including 16 who defaulted for up to 3 years, and 6 whose PP was appropriately stopped by the prescriber based on previous national guidelines. Among the 71 patients still on PP, 57 were classified as fully adherent, while the rest were occasionally or regularly missing their daily penicillin doses.

Penicillin adherence was estimated among patients who had started penicillin and were either still taking it or had spontaneously defaulted at the time of the review (71 + 16 = 87). Overall, about two third (65.5% = 57/87) of patients were estimated fully adherent and thus considered as effectively protected. No significant association was found between adherence and age at presentation, sex, socio-economic level, mother’s education, follow-up time, household crowdness, or the Ross score at presentation (all Fisher Exact p-values > 0.10).

### Clinical and valvular evolution since 1st presentation

The median follow-up time since first presentation was 3 years (IQR[< 1y; 11]). Most of the 111 patients were stable at the time of the review as 93.7% scored 1 to 2 on the Modified Ross/NYHA Score (Table 3). Seventeen patients had undergone cardiac surgery abroad, including 9 mitral valve repairs and 8 mechanical mitral valve replacements treated with lifelong warfarin. Post-operative complications were rare, the main issue being the adherence to the daily dose of warfarin and the monthly monitoring of international normalized ratio (INR), though there was only 1 case of intracranial bleeding, and no thrombotic complications recorded. At the time of review, about half of the patients (59/111 = 53.2%) were stable either without- or after initial antifailure treatment (Table 3). The other half (52/111 = 46.8%) of the patients had regular worsening of their condition, with repeated hospital admissions, including those who had cardiac surgery. No significant association was found between the clinical evolution and sex, age at first presentation, socio-economic level, Ross score at presentation, or adherence to penicillin (all Fisher Exact p-values > 0.10). Clinical evolution was significantly associated with time since first presentation (Exact p = 0.03) with a significant trend of worsening evolution with increasing follow up time (categorized as < 5y, 5–9y, ≥ 10y) (z-test for trend p = 0.012). There was also a significant trend between lower mother education and worsening clinical evolution (z-test for trend p = 0.04).

**Table 3:** Clinical characteristics at review and adherence to penicillin prophylaxis

Characteristics	N	%
Median heart rate, IQR (n = 111) 4 patients with AF	85 [74;92]	
Normal O_2_ saturation	110	99.1
Patients with tachycardia (> 100/min if age > 11; > 120 if < = 11)	10	9
Modified Ross classification		
Class 1	35	31.5
Class 2	69	62.1
Class 3	6	5.4
Class 4	1	0.9
Pathological murmur at auscultation		
No	9	8.1
Yes	102	91.9
Chest auscultation		
Abnormal	11	9.9
Normal	98	88.3
Missing	2	1.8
Oedema lower limbs		
No	107	96.4
Yes (3 grad2, 1 grad3)	4	3.6
Enlarged liver		
No	102	91.9
Yes	9	8.1
Still under penicillin prophylaxis (N = 93)		
No	22	23.7
Yes	71	76.3
Overall estimated adherence to penicillin prophylaxis (N = 87)		
Defaulters and poorly adherent	19	21.8
Regular intake with occasional missed doses	11	12.6
Fully adherent (rare missed dose)	57	65.5
Clinical evolution since diagnosis (n = 111)		
Patient stable without anti-failure treatment (including 7 defaulters)	17	15.3
Good response to initial anti-failure therapy, now stable	42	37.8
Slight worsening, with adjustment of initial treatment, now stable;	19	17.1
Regular worsening with adjustements of antifailure therapy;	10	9
Serious worsening with repeated hospital admissions;	6	5.4
Cardiac surgery	17	15.3

Standard echocardiography results from the 94 patients who didn’t undergo cardiac surgery are summarized in Table 4. All patients had dysmorphic MV features associated mainly with a MV regurgitation (79 = 84.0%). Twenty five patients had MV stenosis mostly severe (64%) and associated with MVR (16/25, 64.3%). More than half of the patients had an AVR (48/94 = 51.1%), 53 (56.4%) had functional TVR, and 28 (29.8%) had mild pulmonary regurgitation. More than a third (34/94 = 36.2%) of the patients had pulmonary hypertension and 13 (13/94 = 13.7%) had impaired systolic function.

**Table 4:** Echocardiography characteritistics at presentation (baseline) and at review (17 patients with cardiac surgery excluded)

Baseline (N = 86)			Review (N = 94)		
Characteristics	N	%	Characteristics	n	%
MV regurgitation (N = 80)			MV regurgitation (N = 79)		
Mild	13	16.2	Mild-	12	15.2
Moderate	15	18.8	Moderate-	12	15.2
Severe	47	58.8	Severe-	55	69.6
Not specified	5	6.2			
MV stenosis (N = 22)			MV stenosis(N = 25) (10 isolated, no MVR)		
Mild	4	18.2	Mild	2	8
Moderate	2	9.1	Moderate	7	28
Severe	15	68.2	Severe	16	64
Not specified	1	4.5			
AV regurgitation (N = 47)			AV regurgitation (N = 48)		
Mild	18	38.3	Mild	11	22.9
Moderate	14	29.8	Moderate	21	43.8
Severe	12	25.5	Severe	16	33.3
Not specified	3	6.4			
AV stenosis (N = 3 = 1 mild & 2 severe)			AV stenosis (n = 1 moderate in 4 valves dysfunction)		
TV regurtation (N = 63)			TV regurgitation (N = 53)		
Mild	19	30.2	Mild	18	33.9
Moderate	22	34.9	Moderate	18	33.9
Severe	20	31.7	Severe (including 1 severe TVS)	17	33.1
Not specified	2	3.2			
Pulmonary valve (PV) regurgitation	17	19.8	Pulmonary valve (PV) regurgitation (all mild)	28	29.8
Mild	12	70.6	Impaired LV systolic function (EF < 55%)	13	13.8
Moderate	2	11.8	Pulmonary hypertension (ePASP > 40 mmHg)	34	36.2
Severe	1	5.9	Pericardial effusion	5	5.3
Not specified	2	11.8	Valvular evolution (vs baseline; N = 73 available)		
Impaired LV systolic function (EF < 55%)	7	8.1	No change	43	58.9
Pulmonary hypertension (ePASP > 40 mmHg)	33	38.4	Initial lesions decreased	13	17.8
Pericardial effusion	3	3.5	Initial lesions worsened	17	23.3

The evolution of valvular lesions could be assessed in 73 patients for whom measurements were available for their first echocardiography. The median time elapsed since first echocardiography was 20 months (IQR[4;112]). Valvular lesions were unchanged in 58.9% (43/73) patients, had worsened in 23.3% (17/73) and regressed in 17.8% (13/73) patients. The valvular evolution was not significantly associated with sex, age at first presentation, severity of initial lesions, clinical evolution, duration of follow-up, adherence to PP (all Exact Fisher p-values > 0.05).

Based on the severerity of valvular lesions, cardiac function and pulmonary hypertension at the time of our review, it was estimated that 77 patients would have been eligible for heart surgery in addition to the 17 who had this opportunity, hence the vas majority (17/94 = 81.9%) of the cardiac surgery need was not covered.

## Discussion

**Fig. 2:**
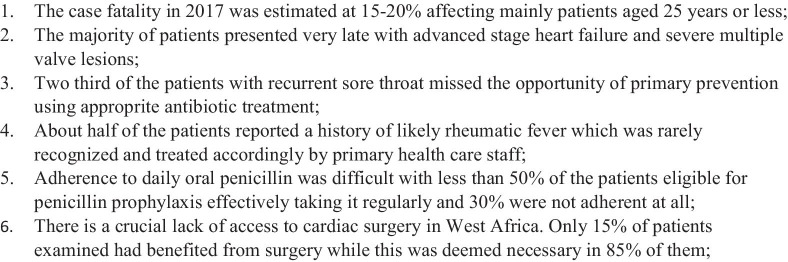
Key learning points from this study

“This is the first publication of the RHD burden in The Gambia, reporting on the clinical and valvular evolution of RHD with related challenges to effective penicillin prophylaxis in a retrospective cohort of 111 RHD patients. While documenting a high burden, we have detected only those who presented to the health care system, largely with symptoms of heart failure suggesting severe disease. These findings suggest that there are many more cases of undetected disease, particularly in adolescents and young adults. For example, in Australia where RHD registration is the most comprehensive in the world, only 19% of cases are severe, 28% are moderate and 53% are mild [21]. Hence in the Gambia the true RHD burden is likely several-fold higher than reported here. This first Gambian report allowed us to highlight key challenges (Fig. 2) to be addressed for the control and elimination of RHD in a generation [12, 22, 23].”

It is important to highlight the high case fatality rate, particularly in young patients, in the face of unavailable cardiac surgery in the country. Our case fatality rate in 2017 was estimated at almost 20% and this was similar (17.8%) to the one reported the same year in a prospective cohort study conducted in Uganda by Okello et al*.* [24]. The REMEDY study which compiled data from 14 LMICs between 2010 and 2012 reported a 2-year case fatality rate of 16.9% [25]. A prior study conducted in Ethiopia in 2005 reported an annual case fatality rate of 12.5% [26]. These data highlight what has been described elsewhere as the”stark reality of rheumatic heart disease” in Africa [27], and other developing countries [28, 29]. Most of our patients (86%) died before their 25th birthday, at a median age of 17 years, showing the high human and socio-economic burden of premature deaths in The Gambia. This calls for the urgent implementation of effective control measures to achieve by 2025 the WHF strategic goal of reducing by 25% RHD mortality in individuals aged < 25 years [8].

The high case fatality rate is consistent with the finding that most patients only seek health care at an advanced stage of heart failure (Ross 3–4) and severe mitral valve lesions are often associated with other valvular lesions. This is a common finding in RHD endemic countries. Indeed, in Uganda, 85% of newly diagnosed RHD patients at the national referral hospital had cardio-vascular symptoms and half of them had complications such as congestive cardiac failure, including 32.7% with pulmonary hypertension [30]. In a later cohort study [24] Okello et al*.* also showed that both morbidity and mortality were significantly higher in patients presenting late with advanced disease. Similar characteristics at presentation were reported by the multi-country REMEDY study [25]. In another report of the REMEDY study [6] atrial fibrillation was documented in 21% of the patients; stroke, systemic embolism, bleeding and infective endocarditis were reported in all countries.

The main reason for late presentation in LMICs is the limited understanding of ARF and/or RHD in the affected communities [31, 32]. In our study, history of recurrent sore throat was reported by more than half of the patients but only a few of them had sought medical treatment, missing the opportunity for primary prophylaxis of ARF. Most patients were unaware of the link between sore throat, ARF and RHD, and believed traditional remedies were more appropriate. This has already been observed in other endemic countries, both within [33–35] and outside [36–38] Africa. Interestingly, about half of our patients reported a history of ARF before developing signs of heart failure and most of them had consulted a health facility. Unfortunately, this opportunity for secondary prophylaxis was missed by the local health staff as only 6 patients were diagnosed and treated for ARF. This is not surprising as ARF is usually underdiagnosed in endemic settings [3, 30, 35, 36]. ARF remains a diagnostic challenge for most clinicians due to the multi-systemic nature of the condition and diversity in presentations [39]. The diagnosis of ARF, given the high occurrence of atypical presentations, is often based on clinical suspicion. Treatment should be started without delay to avoid misdiagnosis, the risk of disease progression and cardiac sequelae. In The Gambia, primary health care staff (mostly nurses) are usually unaware of the moderate to high risk of ARF/RHD in local communities. This explains their low level of suspicion when dealing with aseptic mono-arthritis, polyarthralgia or sub-clinical carditis, a relatively common occurrence [39, 40]. Such a problem has been internationally recognized and the 2015 revision of Jones Criteria now includes echocardiographically diagnosed subclinical endocarditis as a major criterion and recommends echocardiography in all proven and suspect cases [41, 42]. About half of our patients reported signs and symptoms compatible with ARF, offering the opportunity to improve secondary prophylaxis if health staff are adequately trained. This calls for an urgent update of the current medical and nursing curricula to improve ARF case management and provide a more accurate estimation of the ARF/RHD burden.

A few of our patients were diagnosed with heart failure during adulthood. These were predominantly women, about half of them multiparous, with multiple severe valvular lesions, often including mitral valve stenosis, and pulmonary hypertension. This suggests a different pattern of evolution, compared to patients diagnosed during childhood, as RHD lesions may have developed at sub-clinical level very slowly, allowing the heart to progressively adapt to new valvular lesions and cope with related hemodynamic changes until decompensation, *i.e.* after several pregnancies. The physiological hemodynamic changes during pregnancy, including increased stroke volume, heart rate and cardiac output, are poorly tolerated in patients with rheumatic valve disease. Indeed, in LMICs, RHD is the most frequent cardiac condition during pregnancy and the first cause of maternal death [43–45]. A recent international registry study on 390 pregnant women has shown that women with moderate, severe or symptomatic rheumatic mitral valve disease, especially those with severe mitral stenosis, are at very high risk of heart failure during pregnancy, while those with mild or asymptomatic mitral valve disease tolerated pregnancy well [46]. Systematic screening for RHD among pregnant women in LMICs, especially primigravidae, could significantly improve the pregnancy outcomes and the prevention of further valve damage with adequate secondary prophylaxis.

Secondary penicillin prophylaxis is essential for the management of ARF and the prevention of recurrences [7] and intramuscular BPG is superior to oral penicillin. Secondary prophylaxis reduces the severity of RHD by preventing disease progression [47, 48], and even by increasing the rate of regression of mitral regurgitation. Since 2013, our patients were given oral secondary PP although only half of them were taking it as recommended. This is not surprising given that in RHD patients with persistent valvular disease oral penicillin should be taken twice daily at least until the age of 40 years [49]. It is difficult to estimate adherence retrospectively as this may vary over time and patient’s age. Indeed, some patients adhered strictly to the treatment for several years before defaulting. The main reported reasons for low adherence or defaulting among children were either financial constraints (transport costs for monthly reviews, or costs for medicine following regular drug shortages), lack of time or motivation of the parents/caretaker for monthly reviews, or the geographical distance from the clinic. Adherence to monthly BPG injections may not necessarily be better as it has other disadvantages (i.e. pain, fear of anaphylactic shock) [50]. Interventions to improve adherence to secondary prophylaxis should consider several factors, e.g. age, socio-cultural factors, and the health system capacity and may include an active recall system, provision of holistic care, involvement of community health workers and delivery of ARF/RHD health education.

“Although valvular lesions worsened in less than 25% of our patients, such estimation does not include those who died (n=35) and those who underwent surgery (n=17). This proportion can also be explained by the fact that most of our patients presented with already severe valvular disease. Concerning the clinical evolution of the 111 patients, almost half of them showed some degree of worsening since presentation.”

. In our context, this is likely due to the combination of late presentation leading to unfavorable outcomes [24, 51], and poor adherence to secondary prophylaxis. Therefore, in The Gambia BPG should be recommended for both primary and secondary prophylaxis. However, given the past history of sudden deaths and current fear of anaphylactic shock, many health care workers still feel anxious about its use. Therefore, we need substantial health education and training about the safe delivery of intramuscular BPG for both primary and secondary prophylaxis. Experience from other countries have shown that improved delivery of BPG can be achieved and results in good adherence by patients [50, 52, 53].

Cardiac surgery is not available in The Gambia, therefore only a very small proportion of RHD patients can benefit from it abroad with the support of private sponsors. The international non-governmental organization (NGO) “Chain of Hope” (https://www.chainofhope.org/) has been supporting cardiac surgery for Gambian children since 2007 but only 3–5 children on average per year are selected. On a positive note, the recent development of cardiac surgery in neighboring Senegal may improve access in the near future to life-saving interventions for our young Gambian patients.

The study had some important limitations due to the retrospective data collection. First, the study design (hospital-based retrospective cohort) did not allow estimating the RHD burden in the local communities. However, the high number of RHD patients presenting at an advanced stage of heart failure and the fact that some of them are diagnosed during adulthood, particularly multigravidae, suggests a high and largely undetected burden of RHD.

Secondly, the absence of EMRS and specific record keeping for ARF/RHD patients at the Teaching Hospital led to an under-representation of their patients (*i.e.* only 10 *versus* 101 patients from MRCG) while they are both referral centers. However, clinical and valvular characteristics at first presentation, evolution, poor access to cardiac surgery and high mortality are probably similar across institutions.. Adherence to penicillin, as well as to anti-failure treatment, might be slightly worse at ESFTH as compared to MRCG, due to frequent stockouts, requiring patients to buy treatment in private pharmacies outside the hospital.

Selection bias may have occurred as only 111 confirmed RHD patients out of the initial 255 RHD registered patients (43.5%) were examined and the severity of lesions at presentation, adherence to penicillin and evolution over time may have been different among non-included patients. It is difficult to speculate how the bias would have influenced the results as the refusal to participate may be related to severity of disease, *e.g.* severely sick or asymptomatic. Moreover, non traced patients may have died and this would increase the case fatality rate. Nevertheless, our estimated case fatality rate is comparable to other prospective cohort studies in LMICS, suggesting minimal bias, if any. Our case series represents a “survivors cohort” and hence is more representative of surviving patients than of all RHD patients. Therefore, prospective cohort studies are urgently needed to adequately assess the clinical and valvular evolution, and adherence to penicillin prophylaxis and case fatality of RHD.

This study highlights for the first time the devastating consequences of RHD on the health of Gambian adolescents and young adults. Our findings suggest a high and largely undetected burden of disease that is not addressed by appropriate secondary prophylaxis. There is a need for the urgent implementation of an effective national RHD control program to decrease the unacceptably high mortality rate, improve case detection and management, and increase community awareness of this disease. As recommended by the World Heart Federation, The Gambia needs a national RHD registry based on echocardiography screening (including pregnant women), the provision of safe and high quality BPG, and improved access to cardiac surgery in order to achieve the “25 by 25” strategic goal.

## Data Availability

The datasets used and/or analysed during the current study available at MRC Unit and can be made available from the corresponding author on reasonable request.
